# Folate-Functionalized Mesoporous Hollow SnO_2_ Nanofibers as a Targeting Drug Carrier to Improve the Antitumor Effect of Paclitaxel for Liver Cancer Therapy

**DOI:** 10.1155/2018/8526190

**Published:** 2018-11-25

**Authors:** Huiling Lv, Chao Wu, Xuan Liu, Andi Bai, Yue Cao, Wenjing Shang, Lili Hu, Ying Liu

**Affiliations:** Pharmacy School, Jinzhou Medical University, 40 Songpo Road, Linghe, Jinzhou, Liaoning 121001, China

## Abstract

In this study, we prepared PTX-loaded mesoporous hollow SnO_2_ nanofibers conjugated with folic acid (SFNFP) for liver cancer therapy. According to SEM and TEM characterization, SFNF showed a mesoporous hollow structure. The average outer diameter was 200 nm, and the wall thickness was 50 nm. The DSC and XRD study showed that PTX in the channels of nanofibers was present in an amorphous state. The in vitro release experiments demonstrated that SFNF could efficiently improve the dissolution rate of PTX. Both in vitro cell experiments and in vivo antitumor experiments showed that SFNFP could efficiently inhibit the growth of liver cancer cells. Therefore, SFNF is a promising targeting antitumor drug delivery carrier.

## 1. Introduction

In recent years, cancer has become the deadliest disease in the world [1]. Specifically, liver cancer caused 466,100 new cases and 422,100 deaths in China alone in 2015 alone [2]. Due to the difficulties in early diagnosis, rapid progress, and low survival rate, liver cancer has become a serious problem that affects people's health all around the world [3]. Except for surgery, chemotherapy is the most common method for treating liver cancer [4–6]. At present, paclitaxel (PTX) [7], Adriamycin [8], and cisplatin injection [9] are commonly used drugs for liver cancer therapy. Paclitaxel has been recognized worldwide as an effective drug for liver cancer. However, the low solubility of PTX limits its clinical application [10, 11].

Currently, nanoporous materials used for improving the solubility of insoluble drugs and their targeting effects have obtained increasing attention over the world [12–14]; these materials include mesoporous silica nanoparticles [15], mesoporous TiO_2_ nanofibers [16], nanoporous carbon [17], nanoporous anodic titanium dioxide layers [18], poly (*ε*-caprolactone) nanofibers [19], and nanoporous inorganic membranes [20]. The materials can be used to load insoluble antitumor drugs, and the space limitation of the mesoporous structure can limit the crystallization of the insoluble drugs and reduce the drug lattice energy, finally resulting in an improved dissolution rate of the drug [11, 21, 22]. Due to the irregular vascular structure of tumor tissues, the mesoporous nanomaterials can penetrate them, leading to the accumulation of drugs in tumor tissues, which will enhance the antitumor effect and reduce undesirable side effects of the drugs [23–25].

In nanomesoporous materials, mesoporous nanofibers with a two-dimensional structure have been widely used for drug delivery, wound dressings, tissue engineering scaffolds, solar cells, and gas sensors. The preparation methods of nanofibers include electrospinning, drawing, self-assembly, template synthesis, and phase separation [26]. Electrospinning is one of the most widely used methods because it can easily control fiber diameter and morphology [27]. Mesoporous nanofibers have unique advantages as drug carriers, such as their nanodiameter, high specific surface area, high pore volume, and a high drug-loading capacity [28]. Pedro P.G.G et al. prepared PLGA-DNR nanofibers that could significantly improve the release rate of Daunorubicin (DNR) [29], which subsequently helped in the effectiveness of in situ cancer treatment. Sasa Kajic et al. used poloxamer 407 to fabricate nanofibers to increase the solubility of carvedilol [30]. Shi Liu et al. also prepared Dye/M5 and Oxa/M5 loaded with asymmetric multilayer polylactide nanofibers, which could effectively retard the recurrence of liver cancer after surgery [31].

Various targeting ligands such as folic acid, sugars, small molecule inhibitors, and antibodies have been used as carrier modifiers to improve the targeting effect of the nanofibers [32]. As a kind of small molecule that targets ligands, folic acid is well known for its overexpression in liver cancer, lung cancer, and so on. Folic acid has many advantages including easy conjugation to nanomaterials, low immunological response, and favorable tumor tissue accumulation. As an example, Manisha Ahir et al. prepared folate-functionalized CuO nanowires for the treatment of breast cancer [33]. The results demonstrated that FA-CuO nanowires accumulated significantly in tumor tissues compared to that in other types of tissue. Additionally, Sumandeep Kaur et al. used folic acid to modify multiwalled carbon nanotubes (FA-MWCNTs), and the FA-MWCNTs showed excellent potential in cancer targeting and killing the cancer cells [34]. These studies showed that nanofibers functionalized with folic acid can be successfully combined with the folate receptor on cancer cells to obtain a preferable antitumor effect.

In this study, a PTX-loaded, folate-functionalized mesoporous SnO_2_ nanofiber drug delivery system (SFNFP) was prepared. An in vitro dissolution experiment was performed to determine whether SFNFP could improve the dissolution rate of PTX. In vitro and in vivo experiments were carried out to examine the antitumor effect of SFNFP.

## 2. Materials and Methods

### 2.1. Materials

Poly (vinylpyrrolidone) (PVP, Aladdin, M_w_=1,300,000) and 3-aminopropyltriethoxysilane (APTES) were purchased from Aladdin Industrial Corporation (Shanghai, China). N,N-Dimethylformamide (DMF), tin dichloride dihydrate (SnCl_2_·2H_2_O), folic acid, 1-ethyl-3[3-dimethylaminopropyl] carbodiimide hydrochloride (EDC), N-hydroxysuccinimide (NHS), sodium dodecyl sulfate (SDS), dimethyl sulfoxide (DMSO), methanol, acetonitrile, ethanol, and paraformaldehyde were obtained from Sinopharm Chemical Reagent Company, Ltd. (Shanghai, China). Paclitaxel (PTX, purity>99%) was purchased from Xi'an Natural Field Bio-Technique Co., Ltd. (Xi'an, China). Annexin V-FITC Apoptosis Detection Kits, thiazolyl blue tetrazolium bromide (MTT), TritonX-100, bovine albumin V, and trypsin were obtained from Nanjing Jiancheng Bioengineering Institute (Nanjing, China). The SMMC-7721 cell line and H22 cell line were obtained from the National Platform of Experimental Cell Resources (Beijing, China).

### 2.2. The Synthesis of SnO_*2*_-NFs (SNF) and SnO_*2*_-FA-NFs (SFNF)

#### 2.2.1. Preparation of SNF

Before electrospinning, a viscous solution was prepared by dissolving 1.8 g PVP in 10 mL DMF under magnetic stirring for 1 h. Then, 2 g SnCl_2_.2H_2_O was added to the solution and stirred for 3 h. The solution was then transferred into a 1 mL plastic syringe jointed to a pump. The diameter of the needle was 0.1 mm, the flow rate was constant at 50 *μ*L/h, the voltage was 20 kV, and the distance between the needle and the aluminum foil collector was 20 cm. All these experiments were carried out at room temperature. After electrospinning, the electrospun material was scraped from the aluminum foil and then annealed at 500°C for 1 h in the air to removed PVP [35].

#### 2.2.2. Amine Functionalization of SnO_*2*_ Nanofibers (SNF-NH_*2*_)

First, 500 mg of the nanofibers mentioned above was dried by an oil bath (100°C) in a round bottom flask for 30 min. After being cooled down to room temperature, 2 mL of APTES was dissolved in 50 mL ethanol and added to the above system, which was filled with nitrogen. The round bottom flask was then attached to a reflux condenser and refluxed at 77°C for 10 h. The final product was separated by centrifugation and washed 3 times with ethanol followed by vacuum drying.

#### 2.2.3. Folic Acid Conjugation on Amine-Functionalized SnO_*2*_ Nanofibers (SFNF)

First, 25 mg of folic acid was dispersed in 25 mL DMSO by sonication. Then, 12 mg EDC and 10 mg NHS were added to the mixture and stirred for 3 h in the dark. Additionally, 50 mg of SNF-NH_2_, which was dispersed in 1 mL DMSO, was added dropwise to the mixture, and its pH was adjusted to 8 by pyridine. The reaction mixture was then stirred overnight in the dark, washed 2 times by DMSO and 6 times by water, and finally dried to obtain SFNF.

### 2.3. Drug Loading of SNF and SFNF

We chose PTX as the model drug because of its poor water solubility. PTX was loaded into carriers by the adsorption equilibrium method. Next, 100 mg of either SNF or SFNF was incubated with a 1 mL PTX chloroform solution (100 mg/mL) overnight at room temperature. The chloroform solution was removed by centrifugation. Then, we obtained SnO_2_-NFs-PTX (SNFP) and SnO_2_-FA-NFs-PTX (SFNSP). The drug loading capacity was estimated by HPLC (L-2400, HITACHI, Japan).

### 2.4. Characterization

The morphologies and structures of SNF were characterized using scanning electron microscopy (SEM) (JEOL JSM-7001F, Japan) and transmission electron microscopy (TEM) (Tecnai G2F30, FEI, USA), respectively.

### 2.5. X-Ray Diffractometer (XRD) and Differential Scanning Calorimetry Analysis (DSC)

In an attempt to characterize the solid state of PTX, pure PTX, SNF, SFNF, SNFP, SFNFP, and a physical mixture of PTX (PMSNFP) and SFNF (PMSFNFP) were monitored by XRD and DSC assays. We used an X-ray diffractometer (Rigaku Geigerflex XRD, Company, Japan, 30 kV and 30 mA Philips) with Cu-K*α* radiation to record the XRD patterns over a diffraction angle (2*θ* range from 3° to 60° in 0.02° steps), and the scan speed was 5 °/min. DSC was carried out using a differential scanning calorimeter (DSC-60, Shimadzu, Japan) from 30°C to 300°C at 10°C/min under N_2_. The flow rate was 150 mL/min.

### 2.6. N_*2*_ Adsorption-Desorption Analysis

We used a surface area instrument (SA3100, Beckman Coulter, USA) to measure the nitrogen adsorption/desorption isotherms. Before analysis, samples were degassed at 40°C for 12 h to eliminate the effect of moisture. Then, we used the BET method to measure the surface area and the Baret-Joyner-Halenda (BJH) model and the amount of nitrogen adsorption method to measure the pore size distribution and the total volume of the pores, respectively.

### 2.7. In Vitro Drug Release Study

The drug release response from carriers was carried out using a dissolution apparatus (RC-80, Tianjin Guoming Medical Equipment Company, Ltd.) according to the method in the Chinese Pharmacopoeia (2015). We used phosphate buffered saline (PBS, pH=7.4) as the drug release medium; the temperature was 37°C, and the paddle speed was 100 rpm. At the same time, 0.01% SDS was added to the drug release medium. SNFP and SFNFP (both equivalent to 0.7 mg PTX) were added to 500 mL of the medium under stirring. At specific time intervals, 4 mL of medium was transferred into a test tube followed by centrifugation and passing through the 0.22 *μ*m membrane filter. Every time after taking out the medium, 4 mL of fresh PBS was added. The concentration of PTX in the sample at different times was measured by HPLC. We used an Agilent 5 TC-C18 column (250 × 4.6 mm) to analyze the sample. The mobile phase consisted of acetonitrile and water (50/50, v/v) with a flow rate of 1.0 mL/min. All the experiments were repeated in triplicate.

### 2.8. In Vitro Cell Assay

#### 2.8.1. Cell Culture

A cell line of SMMC-7721 was cultured using RPMI-1640, which consisted of 10% fetal bovine serum, 100 U/mL penicillin, and 1% streptomycin, in an atmosphere of 37°C and 95% air with 5% CO_2_. The culture medium was changed every 2 days, and 0.25% trypsin was used to digest the cell during the cell passage.

#### 2.8.2. Cell Viability Assay (SMMC-7721 Cell)

The in vitro cytotoxicity of SNFP and SFNFP was evaluated by an MTT assay on SMMC-7721 cells. SMMC 7721 cells were seeded in 96-well plates at a concentration of 5 × 10^3^ cells per well and incubated for 24 h at 37°C. Then, cells were treated with different concentrations (1,000, 500, 250, 100, 50, 10, and 5 *μ*g/mL) of pure PTX, SNFP, and SFNFP, which were suspended in the culture medium. To determine the biosafety of SNF and SFNF, they were also suspended in the culture medium, and different concentrations of suspensions (1,000, 500, 250, 100, 50, 10, and 5 ng/mL) were added into the 96-well plates. The cells were incubated for 48 h, and then, a 20 uL MTT (5 mg/mL) solution was added into each well and cultured for 4 h in the dark. Next, the supernatant was discarded and replaced with 150 *μ*L dimethylsulfoxide (DMSO) per well to dissolve the violet formazan crystals. After shaking the sample for approximately 10 min in the dark, the absorbance intensity was recorded at 492 nm on a microplate reader (VERSA max, Molecular Devices, Sunnyvale, CA, USA). The cell viability was calculated using the following formula:

(1) $$\begin{array}{c} \text{Cell viability}=\frac{{OD}_{t}}{{OD}_{c}}\times 100\% \end{array}$$

OD_t_ stands for the absorbance of treated cells and OD_c_ stands for that of the control cells.

#### 2.8.3. Cell Uptake Assay

To prove that carriers were ingested into the SMMC-7721 cells, FITC-SnO_2_-FA-NFs (FITC-SFNF) was prepared. FITC was dissolved into 1 mL absolute alcohol, and SFNF was added to the mixture and left to rest for 4 h. The mixtures were centrifuged and dried in vacuum. SMMC-7721 cells were seeded in a plate and cultured for 24 h until 80% confluence. Then, the cells were incubated with FITC-SFNF at 50 *μ*g/mL (dispersed in RPMI-1640) for 1, 2, and 3 h at 37°C. In addition, the culture medium was removed and washed more than three times by PBS. The cells were fixed with 4% paraformaldehyde solution and 1 *μ*g/mL Hoechst 33342 for 20 min and 2 *μ*g/mL rhodamine B for 30 min in the dark at 37°C. Finally, the cells were soaked with 1 mL of PBS at 4°C to preserve or examine immediately by confocal laser scanning microscopy (CLSM).

The cell uptake amount of SNFP and SFNP was further measured by HPLC. The SMMC-7721 cells were incubated with PTX, SNFP, and SFNFP at 50 *μ*g/mL (dispersed in RPMI-1640) for 1, 2, and 3 h at 37°C. Then, the cells were digested and washed with PBS and centrifuged. The collections were dispersed in 1 mL of saline solution under ultrasonication for an hour. Next, 500 *μ*L methyl tert-butyl ether was added, and the sample was vortexed for 5 min to extract PTX. The suspensions were then centrifuged for 20 min at 10,000 rpm, evaporating the organic layer in a vacuum. The sample was redissolved with 30 *μ*L of methanol, and 20 *μ*L of it was analyzed in the HPLC.

#### 2.8.4. Cell Apoptosis Assay

SMMC-7721 cells were seeded in a 6-well plate and incubated for 24 h in a proper atmosphere. Then, pure PTX, SNFP, and SFNFP (suspended in RPIM-1640 medium, equivalent to 10 ng/mL PTX) were added to the cells. After culturing for 48 h, the cells were washed with PBS three times and digested with trypsin. Then, the cells were collected and centrifuged for 5 min at 1,000 rpm and washed by PBS and centrifuged again. The samples were suspended in 500 *μ*L of binding buffer, and 5 *μ*L of Annexin V-FITC and 5 *μ*L of PI were added to the sample via suspension under dark conditions. Flow cytometry was used to detect the cell apoptosis.

#### 2.8.5. Western Blot Analysis

SMMC-7721 cells were seeded in 4 culture dishes and treated with pure PTX, SNFP, and SFNFP at a concentration of 10 *μ*g/mL, and the last dish was the control group. After 48 h, the supernatant was removed, and the cells were washed with PBS three times. Then, 300 *μ*L of lysis buffer was used to collect cells, which was centrifuged for 20 min at 12,000 rpm after using an ultrasonic cell crusher for 30 seconds. The total protein concentration was calculated by BCA assay. Next, 20 *μ*L protein samples were subjected to polyacrylamide gel and transferred to nitrocellulose membranes. Then, the membranes were blocked with 5% nonfat milk for more than 1.5 h and incubated with antibodies at 4°C overnight. After being washed 3 times with TBST, the membranes were incubated with secondary antibodies for 1.5 h. An ECL detection kit was used to visualize the samples, and Quantity one 1-D Analysis Software (Bio-Rad, Hercules, USA) was used to analyze them.

### 2.9. In Vivo Experiments

We chose Kunming mice (8 weeks, weighing 18-22 g, female) purchased from the Laboratory Animal Science Department of Jinzhou Medical University to perform the study.

#### 2.9.1. The Establishment of Ascitic Tumor

An H22 hepatoma cell line was cultured in RPMI-1640 containing 10% fetal bovine serum, 1% streptomycin, and 100 U/mL penicillin in an atmosphere of 37°C and 95% air with 5% CO_2_. Then, the H22 cells (1 × 10^5^) were injected into the enterocoeles of the Kunming mice. After a week, there was significant swelling in the abdomen. We extracted the ascites and diluted them with physiological saline. Next, 1 × 10^5^ H22 cells were injected by subcutaneous inoculation in the unilateral armpit. After one week, we could see an obvious tumor on the mice.

#### 2.9.2. Antitumor Activity of SNFP and SFNFP

When the primary tumor volume was up to approximately 500 mm^3^, 16 mice were randomly assigned to 4 groups, each group having four mice: the untreated control group, PTX group, SNFP group, and SFNFP group. Every mouse was treated with 20 mg of pure PTX per kilogram of weight. Three groups of mice were injected with pure PTX, SNFP, and SFNFP, and the last group was given physiological saline as the control group. The drug was given every 3 days, and administration continued for 18 days. Every time before giving the drug, we measured the weight and the longest diameter and shortest diameter of the tumor. The mice were euthanized after the final administration of the drug, and the tumor weight was measured. The tumor volume was calculated using the following formula:

(2) Volume  of  tumor=longest  diameter×shortest  diameter22

The tumor inhibition rate was calculated using the following formula:

(3) $$\begin{array}{c} \text{Tumor inhibition rate}=\left(\;1-\frac{W_{t}}{W_{c}}\;\right)\times 100\% \end{array}$$

W_c_ stands for the weight of the tumor of the control group, and W_t_ stands for the mean weight of the tumor for each drug treatment group.

### 2.10. Data Processing and Statistical Analysis

The results of all experiments were reported as the mean ± SD. Statistical significance was analyzed by ANOVA for multiple groups and P<0.05 was considered to be statistically significant.

## 3. Results and Discussion

### 3.1. Synthesis and Characterization of SNF

**Scheme 1:**
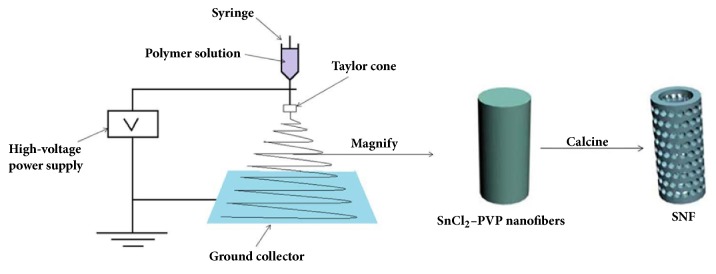
Schematic diagrams for illustrating the synthesis of SNF.

The synthesis of SNF is shown in Scheme 1. The SnCl_2_-PVP nanofibers were prepared by the electrospinning method. During the electrospinning process, a high-voltage power supply, a syringe pump, a syringe filled with the polymer solution and equipped with a metallic needle, and a ground collector were used. When the needle was connected to the high-voltage power supply, an electrostatic field could be formed. The droplets on the needle tip generated and formed under the high voltage a Taylor cone. A single and fast whipping viscoelastic jet was produced from the tip of the Taylor cone and then split into many small jets. The small jets were further stretched in the electrostatic field, ultimately resulting in nanofiber products [36]. The PVP was then removed by calcination to form mesoporous hollow SnO_2_ nanofibers.

**Figure 1:**
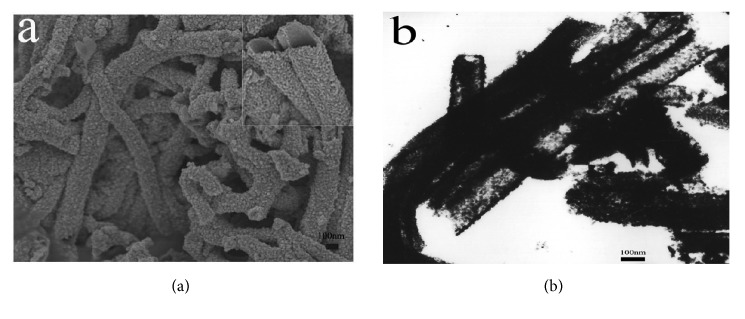
The SEM (a) and TEM (b) images of SNF.

As indicated in Figure 1(a), the SEM image of SNF had a regular nanofiber appearance with a diameter approximately 200 nm. The TEM image (Figure 1(b)) revealed that the SNF showed a mesoporous hollow structure, and the average diameter was consistent with the above conclusion.

To obtain the targeted carrier, SNF was functionalized with folic acid to form SFNF. The structural characteristics of the carrier were further confirmed by the nitrogen adsorption/desorption analysis method. As shown in Table 1, the specific surface area (S_BET_), pore volume (V_t_), and total pore diameter (D_BJH_) of SNF were 26.29 m^2^/g, 0.08 ml/g, and 12.50 nm, respectively. These results indicated that SNF had a relatively high specific surface area and large pore volume. The values of SFNF were 20.49 m^2^/g, 0.06 ml/g, and 11.44 nm because of the connection with folic acid. After being loaded with PTX, the S_BET_, V_t_, and D_BJH_ values for SNFP were 3.96 m^2^/g, 0.02 ml/g, and 18.41 nm, respectively, and the corresponding values for SFNFP were 4.20 m^2^/g, 0.03 ml/g, and 14.59 nm, which were lower than those for SNF and SFNF. The results indicated that PTX was successfully loaded into the SNF and SFNF. The HPLC analysis indicated that the drug loading of SNFP and SFNFP was 18.40% and 22.37%, respectively. Therefore, SFNF was suitable as an antitumor drug carrier.

### 3.2. Characterization of SFNF by FTIR

**Figure 2:**
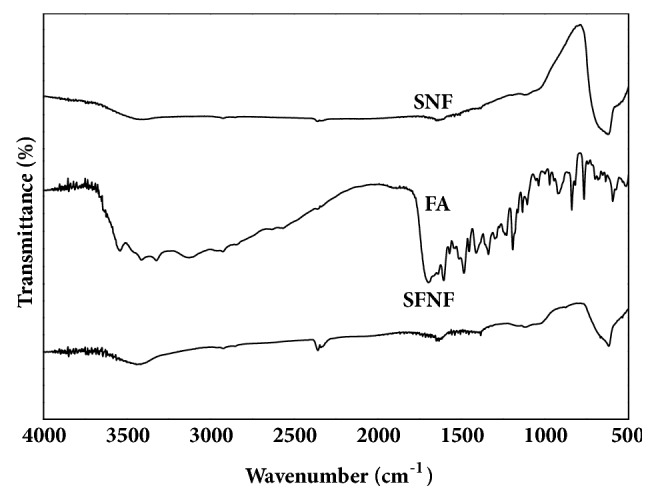
FTIR spectra of SNF, FA, and SFNF.

Modification of SFNF was confirmed by FTIR. The spectra of SNF in Figure 2 exhibited one peak, occurring at 623 cm^−1^, which could be attributed to an Sn-O-Sn bond. Folic acid showed a characteristic peak at 1690 cm^−1^, which was assigned to C=O stretching. In the spectrum of SFNF, the specific peak for C=O was at 1639 cm^−1^, and the peak at 3429 cm^−1^ showed the N-H stretching vibrations, indicating the formation of an amide bond. The above conclusion confirmed the successful binding of FA with SNF.

### 3.3. Solid-State Characterization

**Figure 3:**
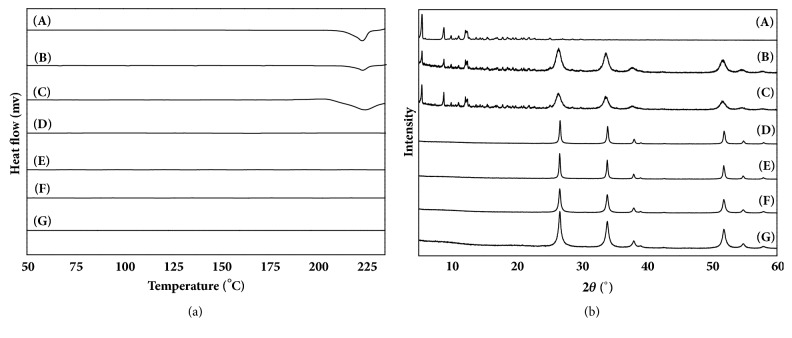
The (a) DSC patterns and (b) XRD patterns of (A) pure PTX, (B) PMSNFP, (C) PMSFNFP, (D) SNF, (E) SFNF, (F) SNFP, and (G) SFNFP.

DSC was used to confirm the solid state of the drug in the carrier. The DSC curves of pure PTX, SNF, SFNF, SNFP, SFNFP, PMSNFP, and PMSFNFP samples are presented in Figure 3(a). The DSC curve of pure PTX produced a single sharp endothermic peak at 223.0°C. The SNF and SFNF did not show any endothermic peak. The endothermic peaks of PMSNFP and PMSFNFP also emerged at 223.0°C, which was attributed to the melting of PTX. These results demonstrated that PTX did not change its physical state in PMSNF and PMSFNF. In comparison, SNFP and SFNFP had no peak at 223.0°C in their thermograms, indicating that PTX was present in an amorphous state in SNF and SFNF.

PXRD analysis was carried out to investigate the state of PTX further. As shown in Figure 3(b), the main diffraction peaks of SNF were distinctive at 26.7°, 34°, 51.86°, 38.1°, and 54.88°; these peaks were assigned to the (110), (101), (211), (200), and (220) planes of SnO_2_, respectively. PTX exhibited intense and typical diffraction peaks at 2*θ* =5.49°, and PMSNFP and PMSFNFP also exhibited a diffraction peak at 2*θ* =5.49°. This peak was attributed to the superimposed peak of pure PTX. In contrast, SNFP and SFNFP did not show any characteristic diffraction peaks. These results proved that PTX was successfully loaded into the channels of SNF and SFNF and was present in an amorphous state.

### 3.4. In Vitro Drug Release Study

**Figure 4:**
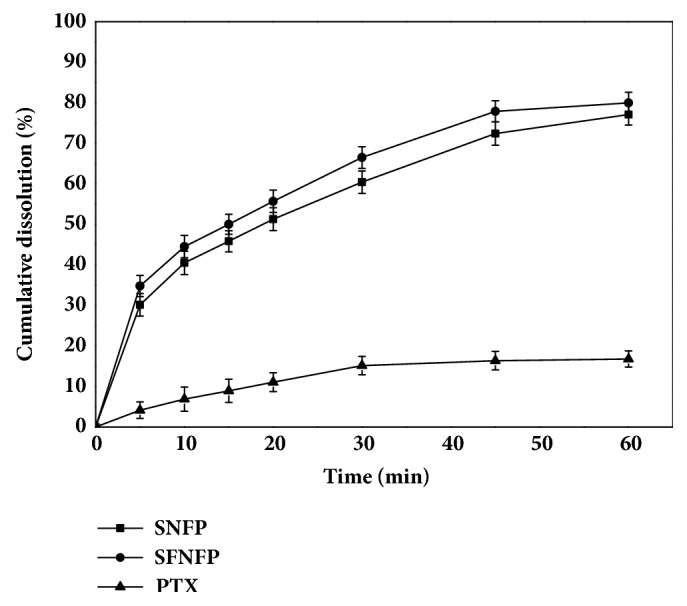
In vitro drug release curves of pure PTX, SNFP, and SFNFP in PBS (pH 7.4) containing 0.01% SDS at temperature of 37°C. Data represented as mean ± SD (n=3).

The dissolution rates of SNFP and SFNFP are shown in Figure 4. Both SNFP and SFNFP showed obviously higher dissolution rates than that of pure PTX. The cumulative dissolution of pure PTX was only 4.15±2.01% in 5 min, while the corresponding amounts of SNFP and SFNFP were 30.16±2.78% and 34.80±2.61%, respectively. When the time reached 1 h, the release of SNFP and SFNFP was 77.09±2.58% and 80.00±2.64%, respectively, compared with only 16.77±2.00% for PTX. The faster release of SFNFP and SNFP was because the mesoporous structure of SFNF and SNF limited the particle size of PTX, PTX was in a noncrystalline state in the carriers, and the surface area of PTX was increased. According to the Noyes-Whitney equation [37], the dissolution rate is directly proportional to the effective surface area of the drug particles. Therefore, SFNF and SNF could effectively improve the dissolution rate of PTX.

### 3.5. MTT Assay

**Figure 5:**
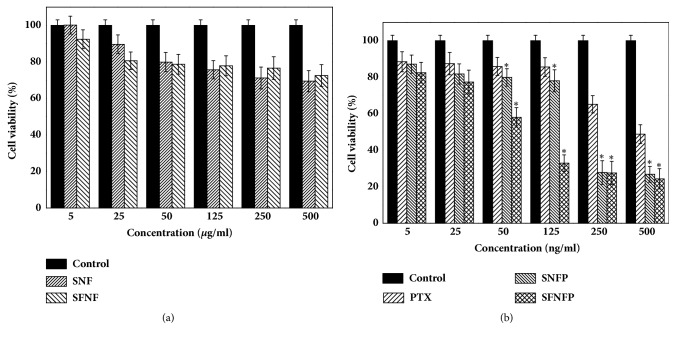
The cell viability of SMMC-7721 cells incubated with (a) SNF and SFNF; (b) PTX, SNFP, and SFNFP for 48 h. Data represented as mean ± SD (n=6).

The biological safety of SNF and SFNF was evaluated by MTT assay. As shown in Figure 5(a), after treatment with SNF and SFNF for 48 h, the viability of the cells was up to 90%. The results indicated that both SNF and SFNF were safe.

The cell viability results of SNFP and SFNFP are shown in Figure 5(b). When the concentration of SNFP and SFNFP was equal to 125 ng/mL of PTX, the cell viability was 78.03±3.45% and 32.91±15.5%, respectively. In comparison, the cell viability of pure PTX was 85.61±3.64%. While the concentration was up to 500 ng/mL, the cell viability of SNFP, SFNFP, and pure PTX was 26.84±18.61%, 24.36±18.84%, and 48.79±28.10%. The SFNFP exhibited the largest inhibition effect compared to that of SNFP and pure PTX. The IC50 (the half maximal inhibitory concentration) of SNFP was 132.16±7.87 ng/mL, and that of SFNFP was 69.76±12.14 ng/mL, while for pure PTX, the value was 623.41±50.17 ng/mL. Therefore, compared with SNFP and PTX, SFNF could effectively inhibit the growth of SMMC-7721 cells. This difference was because FA has a high affinity for the folate receptor on the SMMC-7721 cell membranes. Folate receptors are considered to be overexpressed in liver cancers and can enhance cell endocytosis via the mediation of the folate receptor. SFNFP can enter cells by folate receptor-mediated endocytosis and pass through the organelles by vesicular trafficking; then, PTX can be released into the cell cytoplasm [38]. These findings indicated that SFNFP showed great targeting ability compared with that for SNFP and pure PTX.

### 3.6. Cellular Uptake Assay

**Figure 6:**
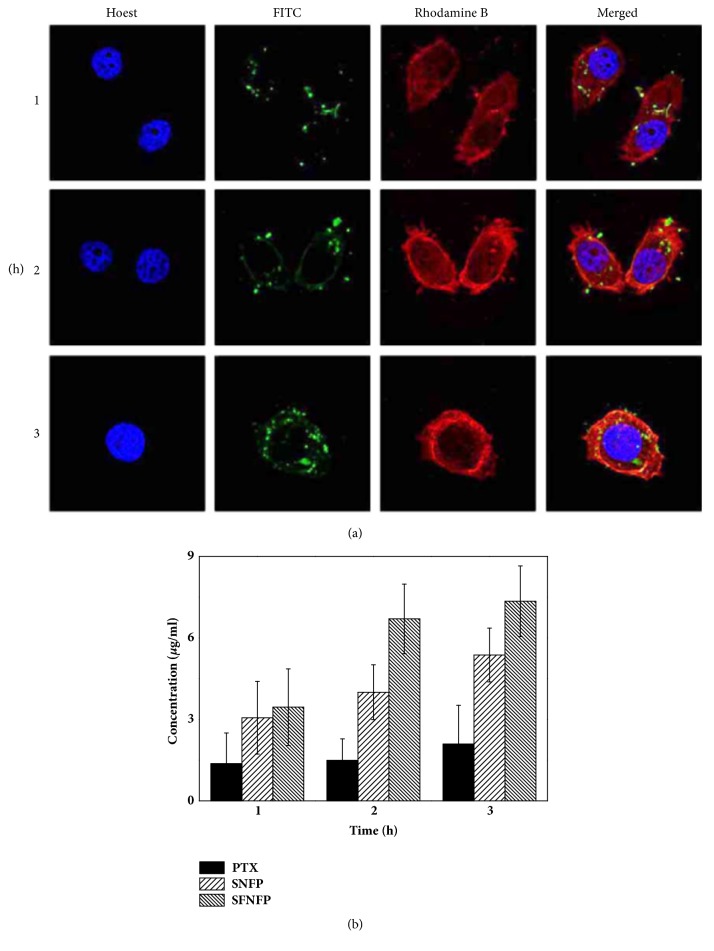
(a) Confocal laser scanning microscopy (CLSM) images of SMMC-7721 cells treated with SFNFP for 1, 2, and 3 h, respectively. (b) The PTX concentration in SMMC-7721cells treated with pure PTX, SNFP, and SFNFP. Data represented as mean ± SD (n=3).

The cellular uptake of the carriers was analyzed by CLSM. The green, red, and blue fluorescence represented the carrier, cytoskeleton, and nucleus, respectively. As shown in Figure 6(a), weak green signals could be seen when cells were treated with FITC-SFNF at 1 h, indicating that the cellular uptake remained low. When the cells were treated for 2 h, the density of the green fluorescence was increased. At 3 h, it could be observed that the green fluorescence appeared stronger than before, which showed that the uptake of SFNF was time-dependent.

To further confirm this conclusion, the uptake of PTX was measured by HPLC. The concentrations of PTX in cells treated with PTX, SNFP, and SFNFP are shown in Figure 6(b). The cellular uptake of SFNFP was 3.59±1.30 *μ*g/mL at 1 h, compared with 3.06±1.34%  *μ*g/mL for SNFP and 1.37±1.13%  *μ*g/mL for pure PTX. When the time reached 3 h, the value of SFNFP was 7.35±1.30%  *μ*g/mL, higher than that of SNFP (6.37±0.99%  *μ*g/mL) and pure PTX (2.09±1.43 *μ*g/mL). It can be observed that the uptake of PTX was time-dependent. The above conclusion indicated that SFNF could exhibit an excellent cellular uptake ability due to the modification with folic acid.

### 3.7. Cell Apoptosis Assay

**Figure 7:**
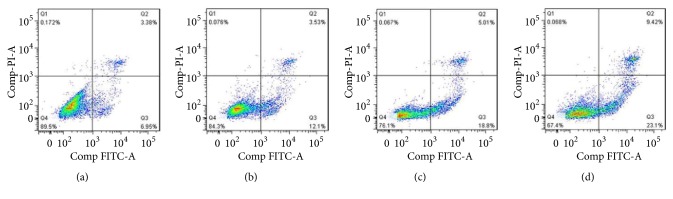
The flow cytometry plots of SMMC-7721 cells treated with (a) the control group, (b) pure PTX, (c) SNFP, and (d) SFNFP.

Quantification of apoptotic cells was carried out by Annexin V-FITC assay using flow cytometry. As shown in Figure 7, the control group showed a high portion of viable cells with a small number of apoptotic cells. The apoptotic rate of SMMC-7721 cells treated with SFNFP was 32.34±1.34%, which was significantly higher than the 15.63±6.16% for pure PTX and 23.81±4.43% for the SNFP-treated group. This result further demonstrated the targeting effect and higher cellular uptake of SFNFP, which led to a high concentration of PTX in SMMC-7721 cells, finally causing the apoptosis of cells.

Western Blot assay was carried out to illustrate cell apoptosis further. We studied the expression of two major components of the Bcl-2 family (Bcl-2 and Bax) and apoptotic markers caspase-3. Caspase-3 is in the cysteine-aspartic acid protease (Caspase) family and serves as a mediator protein in proteolytic degradation during apoptosis. When the proteolytic process conserves aspartic residues, procaspase-3 is transformed from the inactive form to the active form (caspase-3). The Bcl-2 family proteins can participate in mitochondrial apoptosis, mainly regarding aspects of apoptosis inhibition and promotion, playing key roles in cell apoptosis [39–41].

**Figure 8:**
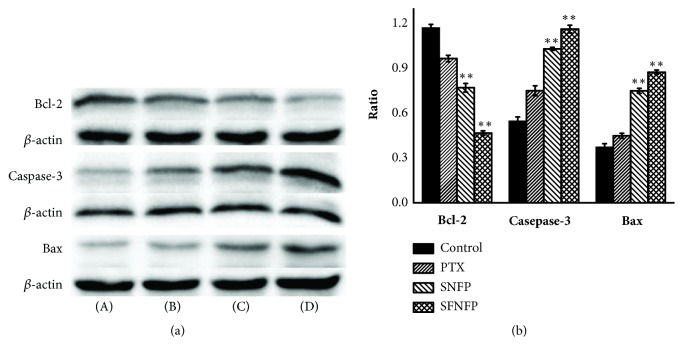
(a) Effect of SFNFP on the expression of Bcl-2, caspase-3, and Bax in SMMC-7721 cells treated with (A) the control group, (B) pure PTX, (C) SNFP, and (D) SFNFP. (b) The statistical analysis of western blot assay. *∗∗P*<0.01 compared with the control group.

The ratios of Bcl-2/*β*-actin for the control, PTX, SNFP, and SFNFP groups were 1.17±0.02%, 0.97±0.02%, 0.77±0.03%, and 0.47±0.02%. The ratio of SFNFP group was significantly lower than that of the other groups. The ratios of caspase-3/*β*-actin were 0.55±0.03%, 0.75±0.03%, 1.03±0.01%, and 1.16±0.02%. The ratios of Bax/*β*-actin were 0.37±0.02%, 0.45±0.02%, 0.75±0.02%, and 0.87±0.02%. These results indicated that the SFNFP group exhibited a higher expression of caspase-3 and Bax than that of the other groups as seen in Figure 8, which meant that SNFP could effectively promote apoptosis due to its targeting ability.

### 3.8. In Vivo Antitumor Effect

**Figure 9:**
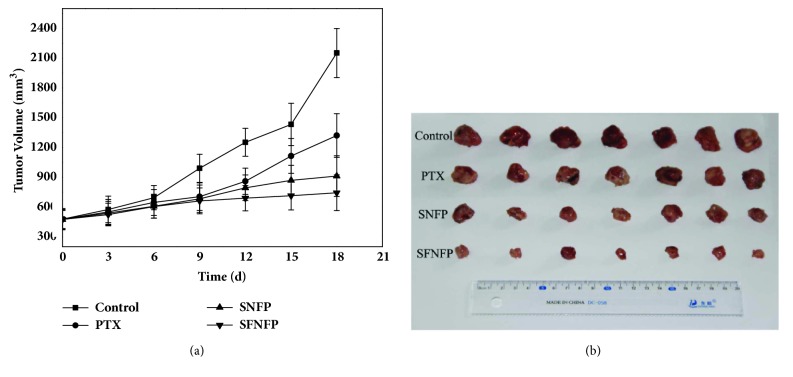
(a) The tumor volume of the control group, PTX group, SNFP group, and SFNFP group. Data represented as mean ± SD (n=3). (b) Images of tumors from each treatment group following excision on day 21.

The antitumor effects of SNFP and SFNFP are shown in Figure 9. The tumor volume of the SNFP and SFNFP groups was significantly smaller than that of the control and PTX groups. At the end of the experiment, the average tumor volume of the control group was 2151.75±40.59 mm^3^. In comparison, the average tumor volume of the PTX group was 1320.50±25.86 mm^3^, and the tumor inhibition rate was 31.12±1.10%. For the SNFP group, the tumor volume was 927.00±19.91 mm^3^, and tumor inhibition rate was 56.91±0.30%. In contrast, the tumor volume and inhibition rate for the SFNFP group were 750±13.64 mm^3^ and 67.06±0.40%, respectively. It was obvious that SFNFP suppressed the tumor growth significantly, better than that of the treatments with SNFP and pure PTX, indicating that SFNFP exhibits good antitumor and targeting abilities.

## 4. Conclusion

In this study, SFNFP was successfully prepared, and PTX was present in an amorphous state in the carrier. The in vitro drug release study showed that SFNFP exhibited immediate release compared to that for pure PTX. In vitro and in vivo experiments showed that SFNFP could effectively inhibit the growth of SMMC-7721 cells and demonstrated a significant tumor regression ability. Generally, these results proved that SFNF could be an ideal drug delivery system for liver cancer therapy due to its incredible targeting antitumor effects.

## Figures and Tables

**Table 1:** Detailed properties of SNF, SNFP, and SFNFP obtained by BET method.

Samples	S_BET_(m^2^/g)	V_t_(ml/g)	D_BJH_(nm)
SNF	26.29	0.08	12.50
SFNF	20.49	0.06	11.44
SNFP	3.96	0.02	10.41
SFNFP	4.20	0.03	9.59

## Data Availability

The data used to support the findings of this study are included within the article.
